# Effects of lipid emulsions on the formation of *Escherichia coli*–*Candida albicans* mixed-species biofilms on PVC

**DOI:** 10.1038/s41598-021-96385-6

**Published:** 2021-08-19

**Authors:** Shanshan Li, Wanshi Duan, Yujie Lei, Zhonghui Wang, Chaojiang Fu, Liang He, Zhenghai Shen, Minjie Li, Ying Chen, Yunchao Huang

**Affiliations:** 1 Department of Thoracic Surgery I, Key Laboratory of Lung Cancer Research of Yunnan Province, The Third Affiliated Hospital of Kunming Medical University (Yunnan Cancer Hospital, Yunnan Cancer Center), No. 519, Kunzhou Road, Kunming, 650106 China; 2 Department of Anesthesiology, The Third Affiliated Hospital of Kunming Medical University (Yunnan Cancer Hospital, Yunnan Cancer Center), Kunming, 650106 China; 3 Department of Thoracic Surgery, Tangdu Hospital, The Second Affiliated Hospital of Air Force Medical University, Xi’an, 710038 China; 4 Department of Palliative Medicine, The Third Affiliated Hospital of Kunming Medical University (Yunnan Cancer Hospital, Yunnan Cancer Center), Kunming, 650106 China; 5 Department of Clinical Laboratory, The Third Affiliated Hospital of Kunming Medical University (Yunnan Cancer Hospital, Yunnan Cancer Center), Kunming, 650106 China

**Keywords:** Biofilms, Pathogens, Experimental models of disease

## Abstract

Patients receiving lipid emulsions are at increased risk of contracting catheter-related bloodstream infections (CRBSIs) in the clinic. More than 15% of CRBSIs are polymicrobial. The objective of this study was to explore the effects of lipid emulsions on the formation of *Escherichia coli* (*E. coli*)–*Candida albicans* (*C. albicans*) mixed-species biofilms (BFs) on polyvinyl chloride (PVC) surfaces and the underlying mechanism. Mixed-species BFs were produced by coculturing *E. coli* and *C. albicans* with PVC in various concentrations of lipid emulsions. Crystal violet staining and XTT assays were performed to test the mixed-species BF biomass and the viability of microbes in the BFs. The microstructures of the BFs were observed by an approach that combined confocal laser scanning microscopy, fluorescence in situ hybridization, and scanning electron microscopy. The study found that lipid emulsions could promote the formation of *E. coli*–*C. albicans* mixed-species BFs, especially with 10% lipid emulsions. The mechanism by which lipid emulsions promote mixed-species BF formation may involve significant upregulation of the expression of the *flhDC*, *iha*, *HTA1*, and *HWP1* genes, which are associated with bacterial motility, adhesion, and BF formation. The results derived from this study necessitate strict aseptic precautions when handling lipid emulsions and avoiding the use of high concentrations of lipid emulsions for as long as possible.

## Introduction

Medical devices for infusion and artificial nutrition are essentially made of plasticized polyvinyl chloride (PVC), and central venous catheters (CVCs) are no exception. CVCs are widely used in clinics to administer parenteral nutrition (PN) due to the high risk of phlebitis^1,2^. However, the risk of catheter-related bloodstream infections (CRBSIs) for patients receiving a long period of PN through CVCs is relatively high due to the presence of lipid emulsions, an indispensable component of PN, which provide vital energy and fatty acids for patients and favour the growth of various microbes, including bacteria and fungi^3,4^.

However, more than 15% of CRBSIs occurring during PN treatment are polymicrobial rather than single infections caused by one microorganism^5^. It is noteworthy that *Candida albicans* (*C. albicans*) is one of the leading pathogens causing CRBSIs associated with high morbidity and mortality. Approximately 27–57% of *C. albicans* infections are associated with other pathogens or opportunistic pathogens^6,7^. *Escherichia coli* (*E. coli*) has gradually surpassed Gram-positive bacteria as the predominant source of CRBSIs^8,9^. *C. albicans* and other pathogens can quickly colonize the surfaces of implanted medical devices and accelerate the formation of biofilms (BFs)^10^. Once BFs form on the surfaces of indwelling medical materials, pathogenic microbes in the BFs can effectively resist immune destruction and antibiotic therapy, leading to the persistence of infections. BFs cause two-thirds of all infections. It has become clear that most BFs are polymicrobial in medical environments^11,12^. According to existing research, clinical biomaterial infections that were polymicrobial had a poorer clinical prognosis than single-species infections, exhibiting twice the mortality rate of single-species infections^13,14^. Several studies have reported that lipid emulsions are associated with an increased risk of contracting a CRBSI. Nevertheless, these studies were limited to studying infections caused by monospecies BFs^15–17^. Few studies have focused on the formation of mixed-species BFs on the surfaces of medical devices. The effects of lipid emulsions on the formation of *E. coli*–*C. albicans* mixed-species BFs on medical catheters are still unknown.

For the above reasons, an in vitro model of a mixed-species BF of *E. coli*–*C. albicans* was constructed on a PVC surface in the present study. We aimed to determine whether the formation of *E. coli*–*C. albicans* mixed-species BFs was significantly altered by clinical lipid emulsions. In addition, we further explored the effects of lipid emulsions on the morphology and architecture of mixed-species BFs. The correlation of the expression of the *flhDC*, *iha*, *HTA1*, and *HWP1* genes with BF formation was also analysed. The *flhDC* gene of *E. coli* encodes transcription factors that initiate flagellar synthesis and enhance the swimming and swarming motilities of the bacterium. The *iha* gene encodes a pathogenic adherence-conferring outer-membrane protein in *E. coli*. *HTA1* has been shown to play a significant role in mediating genomic rearrangement and amplification in *C. albicans*. *HWP1* is directly associated with hypha formation and invasiveness in *C. albicans*.

## Results

### Effects of lipid emulsions on microbial adhesion and mixed-species BF biomass

**Figure 1:**
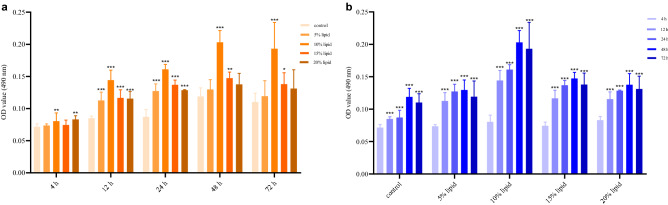
Effect of lipid emulsions on microbial adhesion and mixed-species BF biomass. Compared with the control group at the same time, *P < 0.05, **P < 0.01, ***P < 0.001 (**a**). Compared with the 4 h at the same concentration, *P < 0.05, **P < 0.01, ***P < 0.001 (**b**).

After 4 h, 12 h, 24 h, 48 h, and 72 h of coculture, semiquantitative detection of microbial adhesion and BF biomass on PVC surfaces was conducted in each group. The results showed that the lipid emulsions with various concentrations exhibited greater microbial adhesion and mixed-species BF biomass than those in the control group, which was treated with tryptic soy broth (TSB) medium alone at each time point (*P* < 0.05). The 10% lipid emulsions had the most significant effect, followed by the 15% and 20% lipid emulsions. The detection results for each group from 4 to 72 h showed that the microbial adhesion ability and BF biomass increased with incubation time and were highest at 48 h (*P* < 0.001) (Fig. 1a,b).

### Effects of lipid emulsions on microbial viability in mixed-species BFs

**Figure 2:**
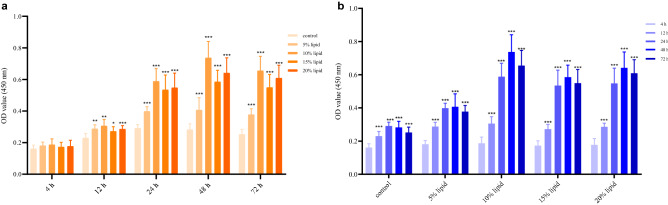
Effect of lipid emulsions on microbial viability in mixed-species BFs. Compared with the control group at the same time, *P < 0.05, **P < 0.01, ***P < 0.001 (**a**). Compared with the 4 h at the same concentration, *P < 0.05, **P < 0.01, ***P < 0.001 (**b**).

After 4 h, 12 h, 24 h, 48 h, and 72 h of coculture, the viability of microbes in mixed-species BFs on the surfaces of PVC pieces was examined by using XTT in each group. There were significant differences between the lipid emulsion groups with various concentrations and the control group at 12–72 h (*P* < 0.05). The 10% lipid emulsion group showed the most significant effect of all groups, followed by the 15% and 20% lipid emulsion groups. The results showed that the microbial viability of each group increased with incubation time and was highest at 48 h (*P* < 0.001) (Fig. 2a,b).

### Thickness and live/dead microbes of mixed-species BFs

**Figure 3:**
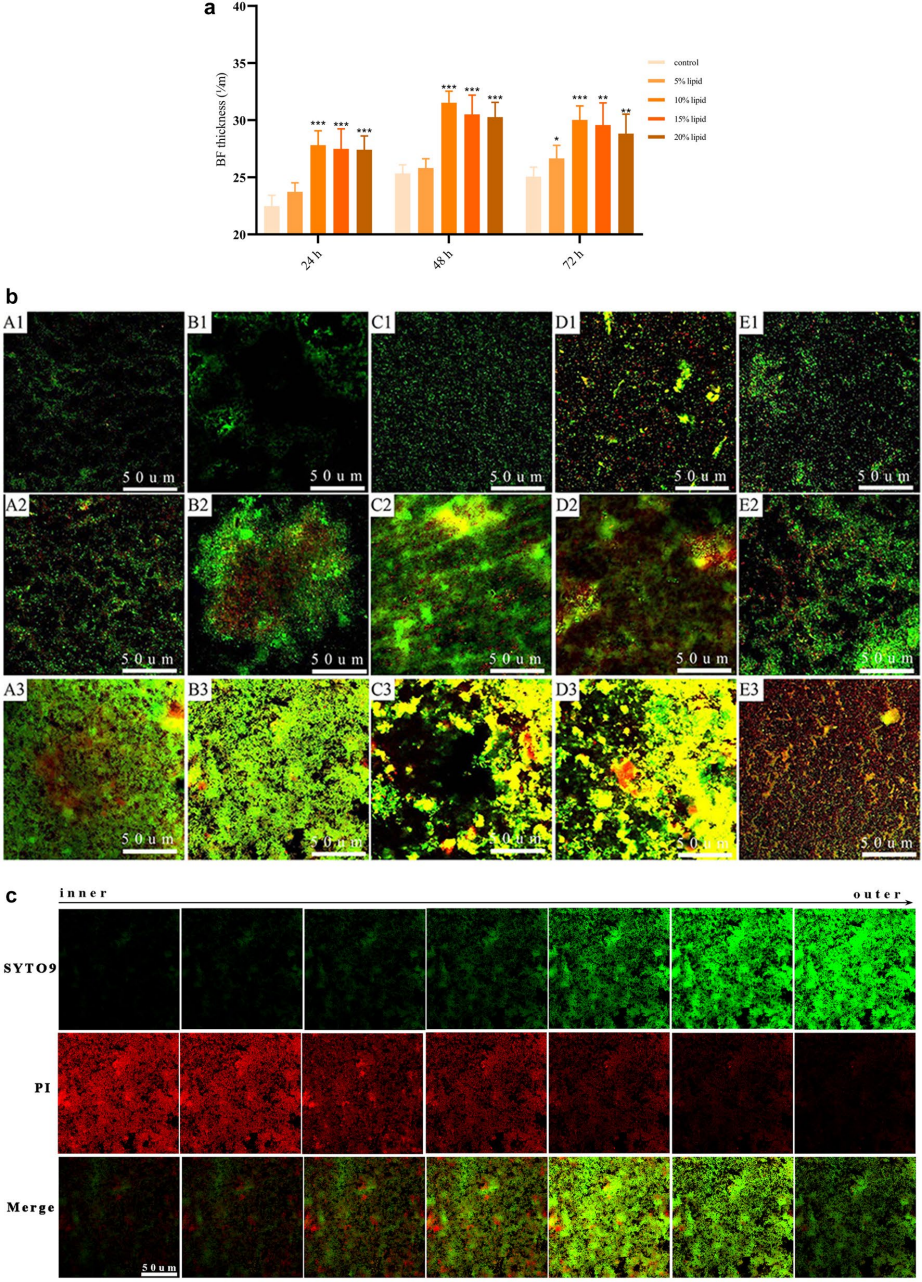
Thickness of mixed-species BFs at 24, 48, and 72 h. Compared with the control group at the same time, *P < 0.05, **P < 0.01, ***P < 0.001 (**a**). Observation of mixed-species BFs by CLSM at 24, 48, and 72 h (**b**). Observation of the live/dead microbes of mixed-species BFs by CLSM (**c**). The images were obtained at × 200 magnification. (**A1**) Control group (24 h); (**B1**) 5% lipid emulsion group (24 h); (**C1**) 10% lipid emulsion group (24 h); (**D1**) 15% lipid emulsion group (24 h); (**E1**) 20% lipid emulsion group (24 h); (**A2**) control group (48 h); (**B2**) 5% lipid emulsion group (48 h); (**C2**) 10% lipid emulsion group (48 h); (**D2**) 15% lipid emulsion group (48 h); (**E2**) 20% lipid emulsion group (48 h); (**A3**) control group (72 h); (**B3**) 5% lipid emulsion group (72 h); (**C3**) 10% lipid emulsion group (72 h); (**D3**) 15% lipid emulsion group (72 h); (**E3**) 20% lipid emulsion group (72 h).

The formation of mixed-species BFs on the surfaces of PVC pieces was found to be a dynamic process. The thickness of the BFs in each group increased rapidly in 24 h of coculture and peaked at 48 h. There was a slight decline at 48–72 h in each group. The mixed-species BFs of the lipid emulsion groups were more complex and denser than those of the control group. The thickness of mixed-species BFs in the 10%, 15%, and 20% lipid emulsion groups was thicker than that in the control group at 24 h, 48 h, and 72 h (*P* < 0.05) (Fig. 3a).

Both live and dead microbes in the mixed-species BFs were observed by confocal laser scanning microscopy (CLSM). The live microbes were stained green by SYTO9, whereas the dead microbes were stained red by propidium iodide (PI). At 24 h, there were only small proportions of dead microbes in the mixed-species BFs in each group. At 48–72 h, the proportions of live microbes decreased, and the proportions of dead microbes increased gradually from the outer layers to the inner layers of the mixed-species BFs (Fig. 3b,c).

### Composition of mixed-species BFs detected by fluorescence in situ hybridization (FISH)

**Figure 4:**
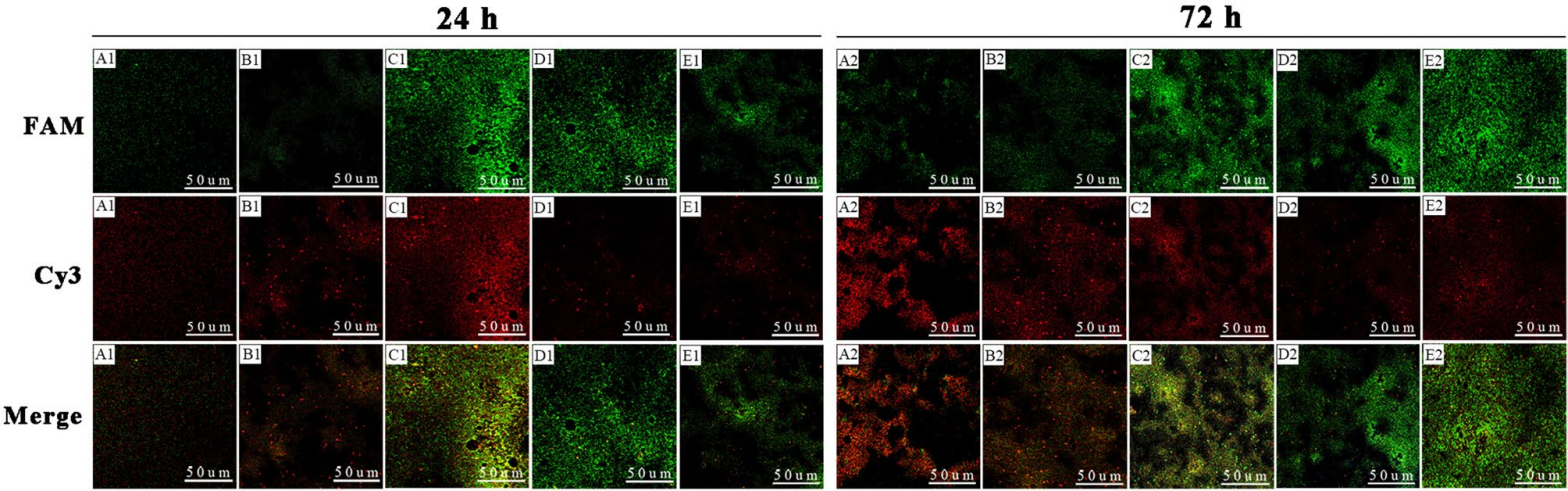
Observation of the composition of mixed-species BFs by FISH-CLSM at 24 h and 72 h. The images were obtained at × 200 magnification. (**A1**) Control group (24 h); (**B1**) 5% lipid emulsion group (24 h); (**C1**) 10% lipid emulsion group (24 h); (**D1**) 15% lipid emulsion group (24 h); (**E1**) 20% lipid emulsion group (24 h); (**A2**) control group (72 h); (**B2**) 5% lipid emulsion group (72 h); (**C2**) 10% lipid emulsion group (72 h); (**D2**) 15% lipid emulsion group (72 h); (**E2**) 20% lipid emulsion group (72 h).

At 24 h, *E. coli* and *C. albicans* grew together in the BFs and formed clumps in the control group. The structure of mixed-species BFs in the lipid emulsion groups was more complex than that in the control group. At 72 h, the BFs were denser and more robust than those at 24 h in each group. After exposure to the 10%, 15%, and 20% lipid emulsions for 72 h, *E. coli* grew around the overlapping and interlaced *C. albicans*, and the mixed-species BFs looked like a “Christmas tree forest”. The “Christmas tree forest”-like appearance indicates the formation of strong BFs. At 24 h and 72 h, in the control group and 5% lipid emulsion group, the proportions of *C. albicans* were greater than those of *E. coli* in the mixed-species BFs. The proportions of these two microbes in the mixed-species BFs were similar in the 10% lipid emulsion group. When the concentration of lipid emulsions exceeded 15%, *E. coli* was obviously dominant in the mixed-species BFs (Fig. 4).

### Microstructure of mixed-species BFs detected by scanning electron microscopy (SEM)

**Figure 5:**
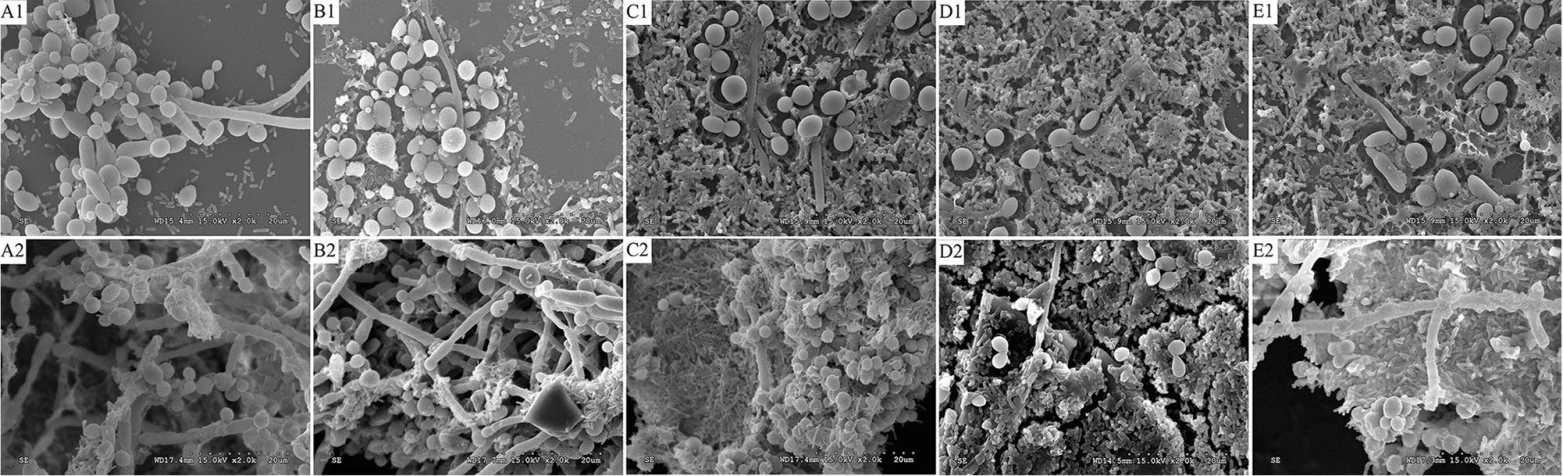
Observation of mixed-species BFs by SEM at 24 h and 72 h. The images were obtained at × 2000 magnification. (**A1**) Control group (24 h); (**B1**) 5% lipid emulsion group (24 h); (**C1**) 10% lipid emulsion group (24 h); (**D1**) 15% lipid emulsion group (24 h); (**E1**) 20% lipid emulsion group (24 h); (**A2**) control group (72 h); (**B2**) 5% lipid emulsion group (72 h); (**C2**) 10% lipid emulsion group (72 h); (**D2**) 15% lipid emulsion group (72 h); (**E2**) 20% lipid emulsion group (72 h).

It was found that *E. coli* adhered to the chlamydospores, pseudohyphae, and mycelia of *C. albicans*. At 72 h, the BFs in each group were more complex and denser than those at 24 h, forming a three-dimensional network structure. The lipid emulsions remained a part of the mixed-species BFs and were attached to the surfaces of bacteria, mycelia and chlamydospores, forming more complex and mature BFs, especially in the 10%, 15%, and 20% lipid emulsion groups. With increasing lipid emulsion concentrations, an increasing amount of *E. coli* was found in the mixed-species BFs. *C. albicans* was closely surrounded by *E. coli*. The growth of *C. albicans* was inhibited when the concentration of lipid emulsions exceeded 15%. There was a significant decrease in the capacity of *C. albicans* to undergo the yeast-to-hypha switch after exposure to 10%, 15%, and 20% lipid emulsions for 72 h (Fig. 5).

### Gene expression by quantitative real-time polymerase chain reaction (qRT-PCR)

**Figure 6:**
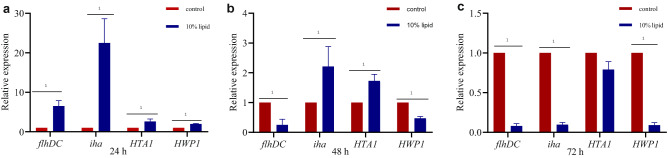
Comparison of qRT-PCR results of gene expression at 24, 48, and 72 h between the control group and the 10% lipid emulsion group, ^1^*P* < 0.05.

Specific differences were noted in the expression levels of various genes between the 10% lipid emulsion group and the control group at various time points. After 24 h of coculture, the expression levels of the *flhDC*, *iha*, *HTA1*, *and HWP1* genes were upregulated in the 10% lipid emulsion group compared with those in the control group. The upregulation of the *iha* gene was the most obvious (*P* < 0.05) (Fig. 6a). The expression of the *flhDC* and *HWP1* genes decreased after 48 h of coculture. The expression of the *iha* gene, which exhibited slight upregulation in the 10% lipid emulsion group after 48 h of coculture, had a similar pattern as that of the *HTA1* gene (*P* < 0.05) (Fig. 6b). After 72 h of coculture, the expression levels of these four genes were downregulated in the 10% lipid emulsion group compared with the control group (*P* < 0.05) (Fig. 6c).

## Discussion

In tumour patients with immune dysfunction and patients who consume glucocorticoids for long time periods, the BFs formed are usually a mixture of bacteria and fungi. *E. coli* and *C. albicans* are often coisolated in cases of BF-associated infections^18,19^. Microbes reside in mixed-species BFs, in which organism growth and metabolism are different from those in monospecies BFs^20,21^. Increased quantity and diversity of the species present increase the complexity of mixed-species BFs and enhance antimicrobial resistance^22,23^. The treatment of mixed-species BFs is more difficult and challenging than that of monospecies BFs. Therefore, research on factors influencing mixed-species BF formation has great clinical significance in reducing CRBSIs induced by lipid emulsions. Our experiments uniquely illustrated differences among various concentrations of lipid emulsions with regard to their effects on *E. coli*–*C. albicans* mixed-species BF formation. These data have the potential to impact the treatment of patients receiving PN.

In our research, PVC was used as the CVC material, and an *E. coli*–*C. albicans* mixture was used as the source of opportunistic pathogen coinfections. TSB medium was used as a supplement for various concentrations of lipid emulsions because it could support both *E. coli* and *C. albicans*^24^. The results of CLSM, FISH, and SEM showed that *E. coli* and *C. albicans* could coexist in TSB medium or lipid emulsions of various concentrations and develop mixed-species BFs on the surface of PVC. Mixed-species BFs were the main cause of coinfection with *E. coli*–*C. albicans* via implanted biomaterials. The results of crystal violet (CV) and XTT assays, and the thickness of the mixed-species BFs showed that microbial adhesion, viability, and mixed-species BF formation were promoted by lipid emulsions, and 10% was the optimum lipid emulsion concentration among the four preset concentrations for the growth and formation of *E. coli*–*C. albicans* mixed-species BFs.

In addition, we used live/dead BacLight bacterial staining and CLSM to visualize the internal structure of mixed-species BFs. This was proven to be a reliable technique for the evaluation of microbial viability in BFs^25,26^. The images revealed that the proportions of live pathogens declined gradually from the outer to inner layers due to the relatively hypoxic and nutrient-deficient environment. Dead pathogens were abundantly located at the bottom and inside of the mixed-species BFs. This was consistent with previous research^10,27^. Nucleic acids released into the extracellular environment by dead pathogens have been demonstrated to contribute to the formation and structural stability of BFs and provide abundant nutrients for live pathogens to support the sustainable growth of BFs^28^. This result suggested that over time, the number of dead cells at the bottom of the BFs increased, and the structure of the BFs became more stable and robust.

Furthermore, by SEM and FISH, we observed a novel phenomenon that wherein *E. coli* was predominant in the mixed-species BFs after exposure to the 15% and 20% lipid emulsions for a long time, and the growth of *C. albicans* was inhibited and transformed from the hypha phase to the yeast phase. However, these results did not conclusively indicate that mixed-species BF formation was impaired when the concentration of the lipid emulsions exceeded 15% because we also observed the “Christmas tree” structure representing strong vitality in these groups. Swindell et al. pointed out that 10% Intralipid was conducive to the germination of *C. albicans* chlamydospores and the growth of mycelia, which was not entirely consistent with our experimental results^16^. The apparent differences between these two studies might be attributed to the BF species and different types of lipid emulsions. However, our findings are supported by other studies. These studies have proven that *C. albicans* has a unique sensitivity to medium-chain fatty acids with carbon chain lengths of C8, C10, and C12, represented by caprylic, capric, and lauric acid, respectively^29,30^. The abovementioned medium-chain fatty acids have been demonstrated to have antifungal effects^31,32^. The lipid emulsions used in our experiments contained medium- and long-chain fatty acids. The carbon chain lengths were C6–24. Moreover, the interactions between species are very complex, ranging from cooperation to competition and even predation^33,34^. *E. coli* can significantly influence candidal growth and induce hyphal death^35^. Interestingly, a study by Cabral et al. demonstrated that *C. albicans* could be killed by coculturing with *E. coli* in vitro and asserted that a soluble factor secreted by *E. coli* resulted in this effect^36^. However, other previous studies showed that *E. coli* could contribute to the colonization and invasiveness and the damaging effects of *C. albicans*^37,38^. These studies may help to explain the differences in the composition and architecture of mixed-species BFs grown in various concentrations of lipid emulsions. The interactions between species and the complex structure of mixed-species BFs can explain why infections caused by mixed-species BFs associated with biomaterials are extremely difficult to treat.

To better elucidate the mechanism underlying *E. coli*–*C. albicans* mixed-species BF formation enhanced by lipid emulsions, *flhDC* and *iha* gene expression in *E. coli* was measured. *flhDC* mainly regulates the biosynthesis of flagella and increases the motility and ability to colonize biomaterials^39,40^. The *iha* gene is the iron-regulated gene A homologue adhesin. The gene encodes a bacterial outer-membrane protein associated with the adhesion of bacteria^41^. In the *E. coli* strains causing primitive acute pyelonephritis, *iha* was associated with higher BF biomass formation^42^. Based on the qRT-PCR results and the functions of *flhDC and iha*, the current study confirmed a potential role of 10% lipid emulsions in promoting the movement and colonization of *E. coli* at the early stage of BF formation. After the initial adhesion was completed, the aggregation capacity of *E. coli* was enhanced by lipid emulsions during the middle stage of BF formation. In addition, *HWP1* and *HTA1* gene expression in *C. albicans* was measured. *HWP1* encodes a fungal cell wall protein required for hyphal development and yeast adhesion to epithelial cells^43^. *HWP1* is directly associated with BF formation in *C. albicans. HTA1* encodes histone H2A of *C. albicans* and reflects the growth and reproductive ability of *C. albicans* cells to a certain extent^44^. The present study showed that *HWP1* and *HTA1* were expressed at a higher level in the 10% lipid emulsion group than in the control group at 24 h, indicating that lipid emulsions further the growth and hypha formation in *C. albicans* only at the early stage of BF formation. This result showed high concordance with SEM results. After 72 h of coculture, the four genes were downregulated in the 10% lipid emulsion group which might be related to nutrient consumption from the medium or most of the microbes in the mature mixed-species BFs entering the dormant stage.

In summary, the effects of lipid emulsions on *E. coli*–*C. albicans* mixed-species BFs were reported for the first time in our study. We demonstrated that lipid emulsions significantly stimulated *E. coli*–*C. albicans* mixed-species BF formation by upregulating the expression of genes related to BF formation. Avoiding the use of high concentrations of lipid emulsions for as long as possible may be helpful in reducing CRBSIs during PN. Finally, further research on the possible mechanism by which lipid emulsions promote the formation of mixed-species BFs is needed to overcome microbial infections.

## Methods

### Microbes, reagents, and equipment

*E. coli* (MC1000) was a gift from the Yale Coli Genetic Stock Center. *C. albicans* (ATCC10231) was purchased from the Institute of Microbiology, Chinese Academy of Sciences. PVC was purchased from Guangdong Kewei (China) Co., Ltd., processed into pieces with sizes of 5 mm × 5 mm or 8 mm × 8 mm, and sterilized by 24 h of formaldehyde fumigation for further experiments. Lipid emulsions (C6–24) were obtained from Fresenius Kabi (China). The TSB medium and XTT bacterial proliferation and cytotoxicity kit were supplied by Huankai (China) Microbial Technology Co., Ltd. The total RNA extraction kit was supplied by Tiangen Biotech (China) Co., Ltd. Primers were synthesized by Sangon Biotech (China) Co., Ltd. The cDNA synthesis kit was procured from Bio-Rad (USA). The FISH kit was purchased from Boxin Biology (China) Co., Ltd. The live/dead BacLight bacterial viability kit was obtained from Life (USA). The S-3000N scanning electron microscope was from Hitachi (Japan), and the FV1000 confocal laser scanning microscope was from Olympus (Japan). qRT-PCR assays were conducted using SuperReal PreMix Plus supplied by Tiangen Biotech (China) and were performed on an ABI 7500 PCR system (USA).

### Culture of microbes and experimental grouping

Standard strains of *C. albicans* and *E. coli* were inoculated on Sarpaul agar plates and MH agar plates, respectively, and incubated at 37 °C for 24 h. Subsequently, a single colony picked from the Sarpaul agar plate or MH agar plate with an inoculation ring was individually inoculated into a test tube containing 5 mL of TSB medium. These tubes were incubated in a constant-humidity oscillator at 37 °C and 200 r/min for 16–18 h. After the cells grew to the logarithmic growth phase, the concentration of the microbial solution in each tube was adjusted to 1.1 × 10^7^ cells/mL using TSB medium in an ultraviolet spectrophotometer for later use. The mixed microbial solution in the study was prepared in a 1:1 ratio; that is, 2 mL of microbial solution was prepared by mixing 1 mL of *C. albicans* yeast solution and 1 mL of *E. coli* bacterial solution. The experiment included five groups: a control group and four groups with various concentrations of lipid emulsions (5%, 10%, 15%, or 20%). The TSB medium was mixed with the 20% lipid emulsions in various proportions to modulate the lipid emulsion concentration to 5%, 10%, 15%, or 20%. The mixed microbial solution with PVC pieces was treated with the above concentrations of lipid emulsions. As a control, samples were prepared similarly with TSB medium. All groups were incubated in a thermostatic incubator at 37 °C with 5% CO_2_.

### Detection of microbial adhesion and BF biomass by crystal violet (CV) staining

Then, 100 µL of the previously prepared lipid emulsions of various concentrations or TSB medium was added to a 96-well cell culture plate, and 10 µL of the mixed microbial solution and a 5 mm × 5 mm PVC piece were added into each well. Six wells were inoculated in each group in each plate. Plates were incubated for 4 h, 12 h, 24 h, 48 h, and 72 h in an incubator at 37 °C to allow *E. coli* and *C. albicans* to adhere to the surfaces of PVC pieces and induce mixed-species BF formation. After coculturing, the medium was removed from the 96-well plates, and 100 µL of PBS was added to wash and remove the floating microbes on the PVC piece three times. After gently washing and discarding the PBS, the BF was fixed with 95% methanol for 30 min. Then, 100 µL of 2% CV dye solution was added to each well after fixation, and the plate was incubated at 37 °C for 30 min. Then, the CV dye solution was discarded, and each well was rinsed with 100 µL of PBS three times. The bound CV was resolubilized in 100 µL of DMSO, and the absorbance was measured at 490 nm on a multifunctional marker to determine the microbial adhesion ability and quantify the biomass of BFs in the experimental wells. Wells containing TSB medium and a PVC piece were similarly processed and used as blank controls. The experiments were repeated three times independently.

### Detection of microbial viability by XTT

The mixed microbial solution was cocultured with various concentrations of lipid emulsions or TSB medium for 4 h, 12 h, 24 h, 48 h, and 72 h as described above and processed for the XTT assay as previously described^45^. Briefly, the PVC pieces in the wells were gently washed three times with cold PBS solution to remove the floating microbes on the PVC pieces. After gently washing and discarding the PBS from the 96-well plates, 100 µL of TSB medium and 20 µL of XTT solution were added to each well, and the plates were incubated at 37 °C for 2 h in the absence of light. Following incubation, the absorbance was measured at 450 nm to detect the viability of microbes in BFs by using a multifunctional marker. Wells containing TSB medium and a PVC piece were similarly processed and used as blank controls. The experiments were repeated three times independently.

### Observation of the thickness and live/dead microbes of mixed-species BFs by CLSM

An 8 mm × 8 mm PVC piece and 100 µL of mixed microbial solution were added to each well of 24-well plates and cocultured with various concentrations of lipid emulsions (2 mL) in an incubator at 37 °C in the experimental groups. As a control, the PVC pieces and mixed microbial solution were treated with only TSB medium. The fluorescent stains for testing microbial viability in the BFs were identified using a live/dead BacLight bacterial viability kit. The protocol for sample preparation for the viability assay is described in detail in the supplementary data (see S1). CLSM observation was carried out using an argon laser. The green fluorescence excitation wavelength was 488 nm, and the emission wavelength was 519 nm. The red fluorescence excitation wavelength was 559 nm, and the emission wavelength was 567 nm. The live and dead microbes on the PVC pieces were evaluated according to the area occupied by the green fluorescence of live microbes and red fluorescence of dead microbes. Each PVC piece was scanned from the inside to the outside to measure the thickness of the BF.

### Observation of mixed-species BF composition by FISH

FISH was used in combination with CLSM to visualize the distribution of the two strains in the mixed-species BFs. The probe sequences of *E. coli* and *C. albicans* designed according to the sequences in GenBank are shown in Table 1. Probes for the 16S rRNA of *E. coli* and *C. albicans* were labelled with 5-carboxyfluorescein (FAM) and cyanine dye (Cy3), respectively. The protocol for sample preparation for FISH is described in detail in the supplementary data (see S2). After FISH and washing, the structure and composition of the mixed-species BFs on PVC pieces were observed by CLSM. The green fluorescence excitation wavelength for FAM was 488 nm, and the emission wavelength for FAM was 519 nm. The red fluorescence excitation wavelength for Cy3 was 559 nm, and the emission wavelength for Cy3 was 567 nm.

**Table 1:** Primer probe sequences.

Strain	Probe sequence	Fluorescent dye
*E. coli* (MC1000)	AGAGAAGCGACCTCGCGAGAGCAAGCGGACCTCAT AAAGTGCGTCGTAGTCCGGATTGGAGTCTGCAACT	FAM (green)
*C. albicans* (ATCC10231)	ACCAGACTTGCCCTCC	Cy3 (red)

### Observation of mixed-species BF microstructure by SEM

The sample preparation method for SEM was mainly as described in the literature^46^. In summary, 8 × 8 mm PVC pieces were removed from the 24-well plates after incubation for 24 h and 72 h as described above, gently washed with PBS solution three times to remove the medium and floating microbes, and then fixed with 2% glutaraldehyde-containing phosphate buffer for 24 h at 4 °C. After primary fixation, the PVC pieces were washed three times with PBS solution for 10 min each to remove fixatives and residual medium. The PVC pieces were then postfixed with 1% osmium tetroxide for 2 h at 4 °C and then dehydrated in an ethanol gradient series (25%, 50%, 75% and 100%) for 20 min. Then, 100% anhydrous ethanol was replaced with isoamyl acetate for 20 min and frozen at − 20 °C following permeation with *tert*-butyl alcohol at 40 °C for 2 h. The samples were scanned by SEM to observe the microstructure of mixed-species BFs after undergoing critical point drying with carbon dioxide (CO_2_) and ion sputter coating with golden brown.

### Quantitation of BF-related gene expression

A 24-well cell culture plate was removed from the control group and 10% lipid emulsion group at 24 h, 48 h, and 72 h, and the BFs were scraped and transferred to 1.5 mL centrifuge tubes. Total RNA was extracted with a total RNA extraction kit for RNA quantitation. Reverse transcription was conducted with the extracted RNA samples by the Bio-Rad iScript cDNA Synthesis Kit. Primers were designed for *flhDC*, *iha*, *HTA1*, and *HWP1*, as well as for 16S rRNA and *ACT1*, which were used as the reference genes. The primer sequences are shown in Table 2. The primers and cDNA template synthesized from the reverse transcription reaction were used for qRT-PCR. The 2^−∆∆Ct^ method was used for comparison of the relative levels of mRNAs^47^.

**Table 2:** *flhDC*, *iha*, 16S rRNA, *HTA1*, *HWP1* and *ACT1* primer sequences.

Target gene	Forward primer (5′–3′)	Reverse primer (5′–3′)
*flhDC*	GCGGTTTGTTGAAAGTGGAT	GATGGCGGTTGACATAAGC
*iha*	ATGATAACCGGGATGGTCAA	CCCATTTGTCGCTCTTCAGT
16S rRNA	GAGAGCAAGCGGACCTCATA	GCAGACTCCATTCCGGACTAC
*HTA1*	ATGTCAGGTGGTAAAGGTAAAG	CTACAATTCTTGAGAAGCCTTAAC
*HWP1*	GGTTGTGAGCCATTAGGGTTA	GGTTGTGAGCCATTAGGGTTA
*ACT1*	ACCACCGGTATTGTTTTGGA	TGGACAAATGGTTGGTCAAG

### Statistical analysis and image construction

SPSS 24.0 statistical software was used for statistical analyses. The experimental data conforming to the normal distribution or meeting the normal distribution after conversion were expressed as the mean ± standard deviation. Analysis of variance (ANOVA) was used for intragroup and intergroup comparisons, and a *t*-test was used for pairwise comparisons. A value of *P* < 0.05 was considered statistically significant. *P* < 0.01 and *P* < 0.001 indicated significant differences. All graphs were generated by GraphPad Prism 8.0.1 (https://www.graphpad.com). Images were converted to appropriate format and resolution using Adobe Photoshop CS6 (https://www.adobe.com).

## Supplementary Information

Supplementary Information.

## Data Availability

All the data in the paper are available.
