# Supplementary material for: Effects of lipid emulsions on the formation of Escherichia coli–Candida albicans mixed-species biofilms on PVC
Source: Sci Rep. 2021 Aug 19;11:16929. doi: 10.1038/s41598-021-96385-6 (PMC8376934; doi:10.1038/s41598-021-96385-6)
Supplement: Supplementary file 1 — Supplementary Information. [file 41598_2021_96385_MOESM1_ESM.pdf]

Effects of lipid emulsions on the formation of *Escherichia coli-Candida albicans* mixed-species biofilms on PVC

Shanshan Li[#1,2], Wanshi Duan[#3], Yujie Lei[1], Zhonghui Wang[2], Chaojiang Fu[4], Liang He[5], Zhenghai Shen[1], Minjie Li[1], Ying Chen[1*], Yunchao Huang[1*]

1. Department of Thoracic Surgery I, Key Laboratory of Lung Cancer Research of Yunnan Province, The Third Affiliated Hospital of Kunming Medical University (Yunnan Cancer Hospital, Yunnan Cancer Center), Kunming 650106, China
2. Department of Anesthesiology, The Third Affiliated Hospital of Kunming Medical University (Yunnan Cancer Hospital, Yunnan Cancer Center), Kunming 650106, China
3. Department of Thoracic Surgery, Tangdu Hospital, The Second Affiliated Hospital of Air Force Medical University, Xi'an 710038, China
4. Department of Palliative Medicine, The Third Affiliated Hospital of Kunming Medical University (Yunnan Cancer Hospital, Yunnan Cancer Center), Kunming 650106, China
5. Department of Clinical Laboratory, The Third Affiliated Hospital of Kunming Medical University (Yunnan Cancer Hospital, Yunnan Cancer Center), Kunming 650106, China

Short running title: Lipid emulsions promote mixed-species biofilm formation

[#] These authors contributed equally to this work

**Correspondence to*: Dr Ying Chen, Yunchao Huang, Department of Thoracic Surgery I, Key Laboratory of Lung Cancer Research of Yunnan Province, The Third Affiliated Hospital of Kunming Medical University (Yunnan Cancer Hospital, Yunnan Cancer Center), NO.519, Kunzhou Road, Kunming, 650106, China.

E-mail: 36410008@qq.com; huangych2001@aliyun.com.

S1: Protocol for sample preparation for the live/dead microbial viability assay:

1. BF formation: One PVC piece and 100 µL of mixed microbial solution were added to each well of 24-well plates for coculturing with various concentrations of lipid emulsions (2 mL) for 24 h, 48 h, or 72 h in an incubator at 37 ℃ in the experimental groups. In the control group, the PVC piece and mixed microbial solution were treated with only TSB medium.

2. The two dye components provided with the live/dead BacLight bacterial viability kit were mixed at a ratio of 1:1. One dye was SYTO9, which produced green fluorescent staining, and the other was propidium iodide (PI), which produced red fluorescent staining.

3. Then, 30 µl of the mixed dye was added to 10 mL of ddH_2O and the solution was kept away from light.

4. The PVC pieces were removed after coculturing for 24 h, 48 h, or 72 h, gently washed with normal saline three times to remove the floating microbes, and placed in new 24-well plates.

5. The PVC pieces were immersed in the fluorescent dye mixture and stained for 20 min at room temperature in the absence of light. After absorbing excess fluorescent dye, the PVC pieces were washed with normal saline to remove unbound dye.

6. Each PVC piece was observed by CLSM.

S2: Protocol for sample preparation for FISH:

1. BF formation: One PVC piece and 100 μL of mixed microbial solution were added to each well of 24-well plates for coculturing with various concentrations of lipid emulsions (2 mL) for 24 h or 72 h in an incubator at 37 ℃ in the experimental groups. In the control group, the PVC piece and mixed microbial solution were treated with only TSB medium.

2. Ageing at room temperature: The PVC pieces were removed at each time point (carefully observing the front and back while keeping the BF face up), placed into PBS solution and rinsed gently three times. Then, the pieces were left at room temperature for 16 h. The BF was fixed on the PVC through BF ageing.

3. Fixation and dehydration: The PVC piece was fixed on the centre of a glass slide with Fixogum rubber glue, 20 μL of lysozyme was dropped on the surface of each PVC piece, the glass was covered to ensure that the lysozyme was in contact with the whole surface of the BF, and the slide was placed at 32°C for 10 min. The slides were removed and fixed in 70% ethanol for 2 min and then air dried.

4. Probe denaturation: 2 μL of each of two probes and 18 μL of probe diluent for each sample were placed in a water bath at 73 ℃ for 5 min without light and then removed and placed on ice for 5 min.

5. Hybridization: Denaturing probes were added to the surface of the BF, and a cover glass was used to cover the whole BF to ensure that the probes were in contact with the whole BF. The cover glass was sealed by adding Fixogum rubber glue around the cover glass. After treatment, the glass slide was placed in a water bath at 73°C for denaturation for 3 min and placed in a box overnight at 46°C.

6. Washing: The Fixogum rubber glue and cover glass were carefully removed with tweezers, and the glass slide was placed into preheated 48°C washing buffer for 30 min and then washed twice for 10 min each.

7. Desalination: The slide was washed with deionized water at 4 ℃ for desalination and dried quickly.

8. The samples were observed by CLSM with suitable lasers.
